# From Computer-Assisted Surgery to Extended Reality: Research Trends, Global Collaboration, and Future Directions in Craniomaxillofacial Surgery

**DOI:** 10.3390/cmtr19030033

**Published:** 2026-07-29

**Authors:** Neha Sharma, Sylvia Kempter, Jokin Zubizarreta Oteiza, Florian M. Thieringer

**Affiliations:** 1 Clinic of Oral and Cranio-Maxillofacial Surgery, University Hospital Basel, 4031 Basel, Switzerlandjokin.zubizarreta@unibas.ch (J.Z.O.); florian.thieringer@usb.ch (F.M.T.); 2 Medical Additive Manufacturing Research Group (Swiss MAM), Department of Biomedical Engineering, University of Basel, 4123 Allschwil, Switzerland

**Keywords:** augmented reality, mixed reality, virtual reality, extended reality, craniomaxillofacial surgery, computer-assisted surgery, surgical planning, research trends, science mapping, bibliometric analysis

## Abstract

**Background**: Extended reality (XR) technologies, including augmented reality (AR), mixed reality (MR), and virtual reality (VR), are increasingly used in craniomaxillofacial (CMF) surgery; yet, no study has systematically mapped the global development of this research. This study provides the first comprehensive evidence mapping of XR and related computational technologies in CMF surgery, identifying research trends, collaborative networks, and future directions. **Methods**: A systematic search of the Web of Science (WoS) Core Collection (data extracted April 2025) combined XR terms (AR, MR, VR, image-guided surgery, and computer-assisted surgery) with CMF surgery terminology. After applying the inclusion criteria, 778 English-language articles published between 1988 and 2024 were analyzed using performance analysis and science mapping tools, including HistCite and VOSviewer. **Results**: Publication output grew exponentially, accelerating markedly after 2010. The *Journal of Cranio-Maxillofacial Surgery* led in both volume and internal citation impact. The USA, Germany, and China accounted for most of the output, with Germany showing a disproportionately high internal citation impact relative to its publication volume. Three developmental phases were identified: foundational computer-assisted surgery (1990s–2000s), technological development and validation (2000s–2010s), and clinical application optimization centered on AR and VR (2010s–present). Co-authorship networks revealed strong regional clusters with limited cross-cluster connectivity. No publications were identified from African countries other than Egypt. **Conclusions**: XR research in CMF surgery has grown substantially over 36 years, with a clear shift toward AR and VR applications. Geographic concentration limited international collaboration, and a focus on technical rather than patient-centered outcomes remains a key challenge. Future work should prioritize global participation, comparative clinical evidence, and patient-reported outcomes to support wider surgical adoption.

## 1. Introduction

Craniomaxillofacial (CMF) surgery encompasses a wide range of complex procedures, from orthognathic surgery and maxillary/mandibular reconstruction to orbital trauma repair and skull base surgery [1]. These procedures rely heavily on spatial understanding, requiring surgeons to translate two-dimensional (2D) imaging into precise three-dimensional (3D) operative plans in anatomically demanding environments [2,3]. Extended reality (XR) technologies, such as augmented reality (AR), virtual reality (VR), and mixed reality (MR), have emerged as tools that directly address this challenge. AR overlays digital anatomy onto the surgical field in real time, for example, through navigation platforms used for intraoperative localization during CMF and head and neck procedures, or through projection-based systems that improve the accuracy of surgical markings. VR supports immersive preoperative planning and surgical rehearsal, as in the planning of complex CMF reconstructions, and MR enables interactive engagement with 3D anatomical data within the real operative environment, allowing surgeons to manipulate holographic reconstructions of patient anatomy while remaining aware of their physical surroundings [4,5,6]. These capabilities make CMF surgery a particularly relevant domain for XR development and adoption [7,8].

Since their introduction to surgical practice in the 1990s, XR tools have evolved considerably alongside advances in computing power, display technology, and medical imaging [9,10]. In CMF surgery, AR has been used for intraoperative navigation and implant positioning, VR for surgical planning in reconstructive and oncologic cases, and immersive simulation platforms for surgical education [11,12,13]. Evidence of clinical benefit is beginning to accumulate across these modalities. A retrospective comparison of AR-based navigation, individualized templates, and freehand technique in mandibular angle osteotomy found significantly better accuracy with AR navigation than with the freehand approach [14]. A randomized controlled trial of immersive VR training for orthognathic surgery reported improved trainee confidence and knowledge compared with conventional training methods [15]. A comparative study of MR-based occlusion setting in orthognathic surgery found that surgical occlusions set using mixed reality were comparable to those achieved with the conventional physical gold-standard method [16]. This mirrors the earlier trajectory of computer-assisted surgery, an established precursor to XR in this field, where techniques such as computer-aided design/computer-aided manufacturing (CAD/CAM)-guided fibula free flap reconstruction now have a more mature evidence base, including meta-analytic support for shorter ischemia and operative times [17] and favorable institutional cost profiles relative to the conventional freehand technique [18].

Research evidence specifically for XR applications remains in an earlier stage by comparison, and clinical validation is not yet uniform across the field, in part because research relevant to XR in CMF surgery is scattered across oral and maxillofacial surgery journals, biomedical engineering publications, computer-assisted radiology journals, and applied sciences [19,20]. For a surgical team or department, it is difficult to judge which applications are supported by robust, reproducible evidence and which remain at an early proof-of-concept stage. As XR becomes part of a broader digital surgical ecosystem alongside CAD-CAM workflows, point-of-care 3D printing, and robotic assistance, understanding where research in this field currently stands is increasingly relevant to its continued development [1,21,22].

This study addresses that gap by systematically mapping XR research and its computational precursors in CMF surgery. The study objectives are to (1) describe publication growth and journal distribution over time; (2) identify the leading countries, institutions, and authors; (3) map collaborative networks and geographic patterns; (4) trace the evolution of research themes through citation and keyword analysis; and (5) identify priorities for future research, collaboration, and clinical translation. Together, these aims are intended to provide clinicians, researchers, and surgical units with a practical overview of where the field has come from and where it needs to go next.

## 2. Materials and Methods

### 2.1. Data Sources and Search Strategy

Data were collected from the Web of Science (WoS) Core Collection database on 5 April 2025. Topic searches (TS) were used to retrieve publications containing terms in titles, abstracts, and author keywords, combined with Boolean operators (AND, OR).

The following search string was applied:

TS= (“augmented reality” OR “AR” OR “mixed reality” OR “MR” OR “virtual reality” OR “VR” OR “image-guided surgery” OR “computer-assisted surgery”) AND TS= (“craniomaxillofacial surgery” OR “craniofacial surgery” OR “maxillofacial surgery” OR “facial reconstruction” OR “skull base surgery” OR “orthognathic surgery” OR “mandibular reconstruction” OR “midface surgery” OR “jaw surgery” OR “head and neck surgery” OR “orbital surgery” OR “cranial surgery” OR “facial trauma surgery”).

The search string included “image-guided surgery” and “computer-assisted surgery,” along with AR, MR, and VR terms. This was intentional as many early CMF publications describing what is now recognized as XR used these broader descriptors before AR, MR, and VR became standard surgical terminology. Excluding them would have omitted foundational literature that directly shaped current XR practice. Throughout this study, the term “XR” refers to the full technological continuum, from early computer-assisted navigation to contemporary AR, MR, and VR platforms. Standalone abbreviations were included in the search to capture publications using abbreviation-only terminology, and any irrelevant results introduced by this approach were removed during the data refinement step described below.

### 2.2. Data Refinement and Inclusion Criteria

After the initial search, results were refined using WoS Citation Topics Meso classifications. This WoS feature assigns each publication to a subject area based on its citation relationships with other documents in the database, irrespective of journal category. Publications assigned to subject areas outside the scope of this study, including nursing, endocrinology, anesthesiology, cardiology, dermatology, polymer science, and metallurgical engineering, were excluded. This step also addressed any false-positive results introduced by abbreviated search terms.

Publications were included if they met all the following criteria: original research or review articles published in English, indexed in the WoS Core Collection, and relevant to XR technologies or computer-assisted surgery in CMF surgery. No date restriction was applied; the search covered all years available in the database up to the extraction date.

### 2.3. Analytical Framework

The analysis followed established approaches in bibliometric research, combining performance analysis with science mapping [23,24]. Performance analysis examined publication output over time, journal distributions, geographic contributions, institutional affiliations, author productivity, and funding patterns.

Science mapping explored the structure and development of knowledge in the field using four complementary methods. Citation analysis identified influential publications and assessed their impact using the metrics in Table 1. Bibliographic coupling identified pairs of publications that share references, highlighting methodological and topical similarities in recent work. This approach is particularly useful for mapping current research directions because it does not rely on future citation accumulation. Keyword co-occurrence analysis examined which terms co-occur across publications, tracking shifts in research focus over the study period. Co-authorship analysis mapped collaboration patterns at the author and country levels.

### 2.4. Data Visualization and Analysis

Descriptive statistics and tables were produced in Microsoft Excel (Office 365). Citation analysis was conducted using HistCite (v.12.03.17). Network visualizations, including co-citation maps, bibliographic coupling networks, keyword co-occurrence maps, and collaboration networks, were generated in VOSviewer (v.1.6.20).

## 3. Results

### 3.1. Database Search and Study Selection

**Figure 1:**
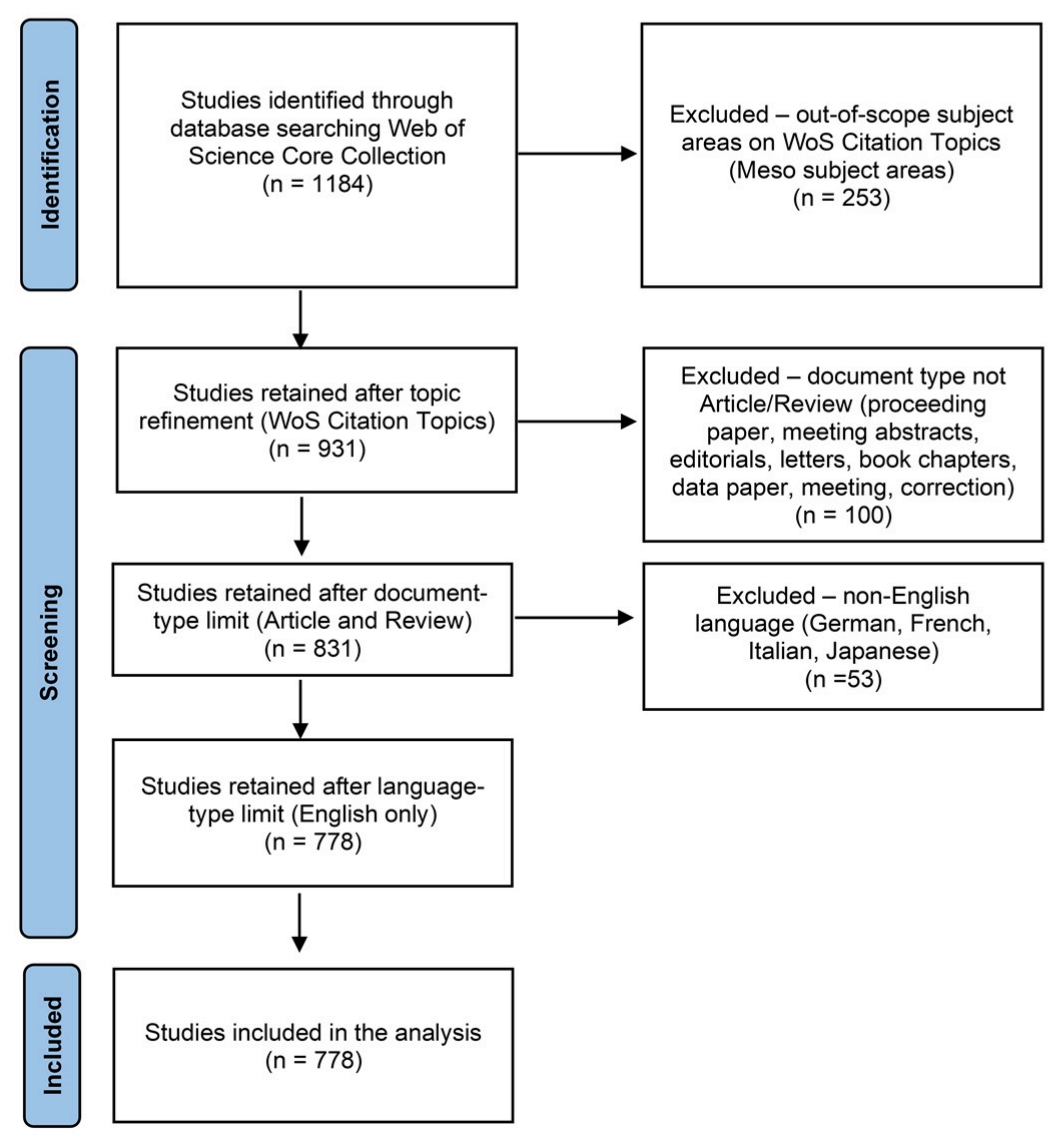
Flow chart of the literature screening and study selection process.

The initial WoS Core Collection search identified 1184 publications. Topic-based refinement using WoS Citation Topics (Meso level), which excluded publications assigned to subject areas outside the scope of this study, removed 253 records and yielded 931 publications. Restricting the results to original research and review articles excluded a further 100 records (proceedings, meeting abstracts, editorials, letters, book chapters, data papers, meeting papers, and corrections), leaving 831. Excluding non-English publications removed 53 records, giving a final dataset of 778 articles. The selection process is shown in Figure 1.

### 3.2. Performance Analysis

#### 3.2.1. Annual Publication Trends

**Figure 2:**
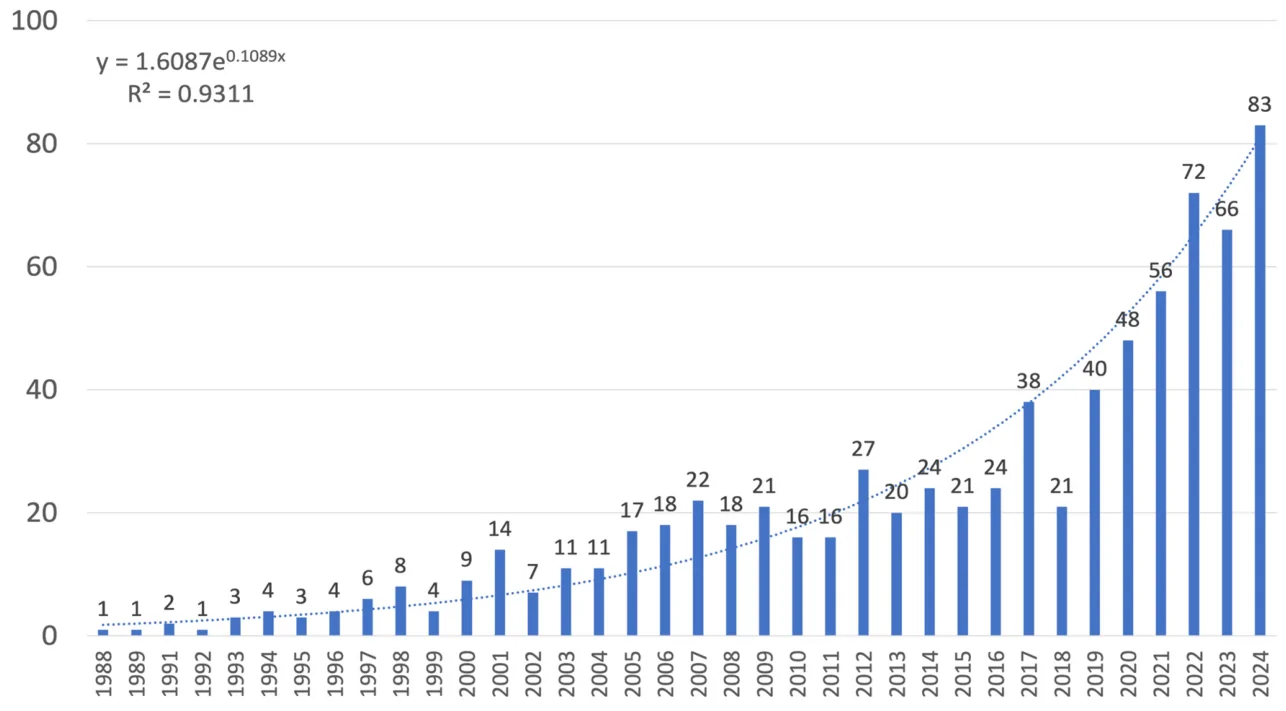
Annual publication output in extended reality (XR) and computer-assisted surgery research in craniomaxillofacial (CMF) surgery from 1988 to 2024.

The earliest publication in the dataset dates to 1988. Output remained low through the 1990s and early 2000s, then gradually increased through the 2000s. A clear acceleration in publication volume is evident from around 2010 onward, continuing through 2024, with year-to-year fluctuations throughout the period (Figure 2). Data for 2025 were excluded as only four months were available at the time of extraction.

#### 3.2.2. Journal Distribution and Impact

The ten most productive journals collectively accounted for 282 publications (36.2% of total output), all of which were specialist journals in oral and maxillofacial surgery, craniofacial surgery, or computer-assisted surgery (Table 2). The *Journal of Cranio-Maxillofacial Surgery* led by publication volume with 56 articles (7.2%), followed by the *International Journal of Oral and Maxillofacial Surgery* (47 articles, 6.0%) and the *Journal of Craniofacial Surgery* (46 articles, 5.9%). The publication volume and the 5-year journal impact factor did not consistently align. The *Journal of Clinical Medicine* ranked tenth by volume (13 articles) but had the highest 5-year impact factor among the top ten (3.4). The *International Journal of Computer Assisted Radiology and Surgery* ranked fourth in volume (25 articles) with an impact factor of 3.1.

#### 3.2.3. Geographic Distribution

**Figure 3:**
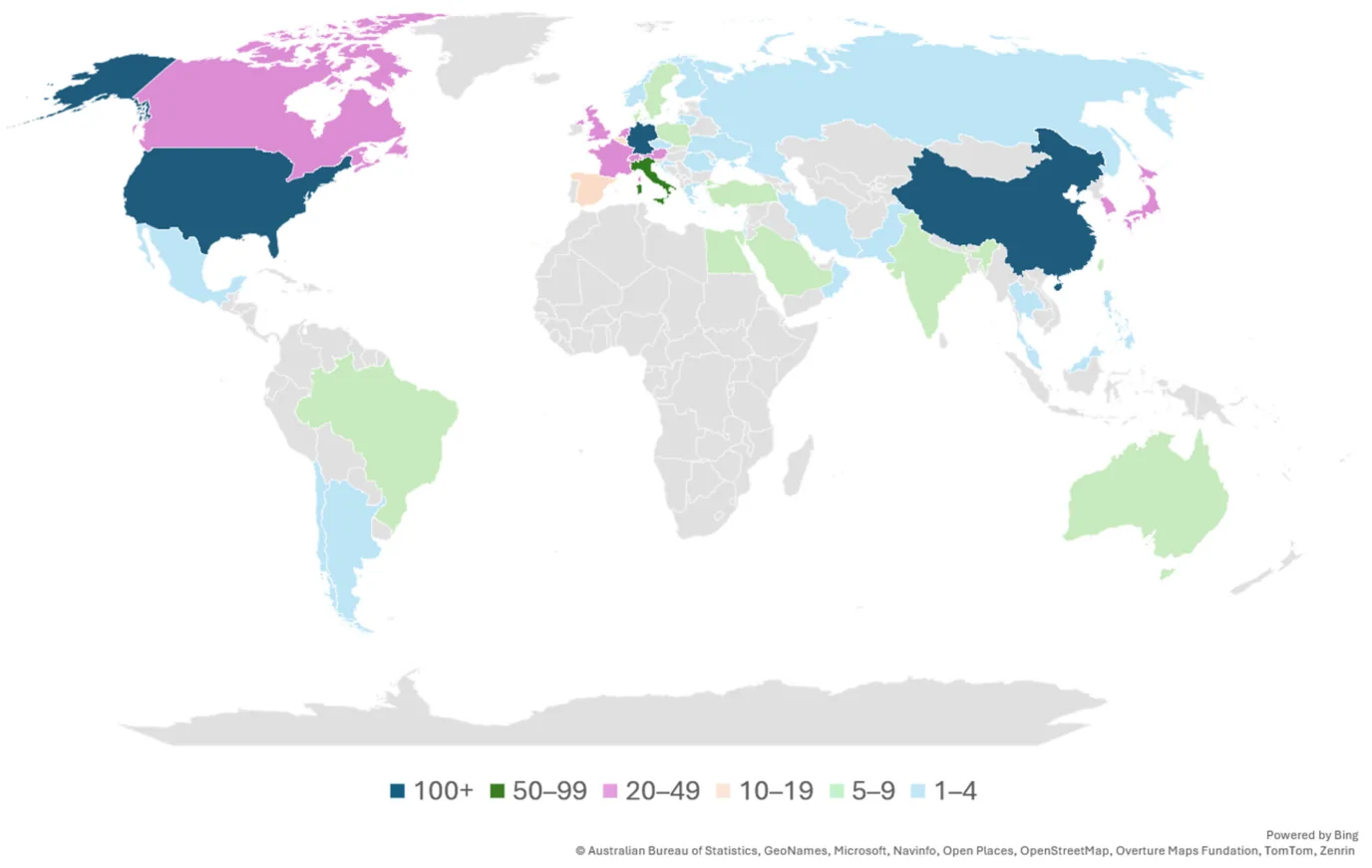
Global distribution of publication output in craniomaxillofacial computer-assisted surgery and extended reality (XR) (Web of Science Core Collection, 1988–2024; *n* = 778). Countries are shaded by the number of publications using discrete classes (1–4, 5–9, 10–19, 20–49, 50–99, and ≥ 100), with darker shades indicating higher output. Countries with no recorded publications in the dataset are shown in beige.

**Figure 4:**
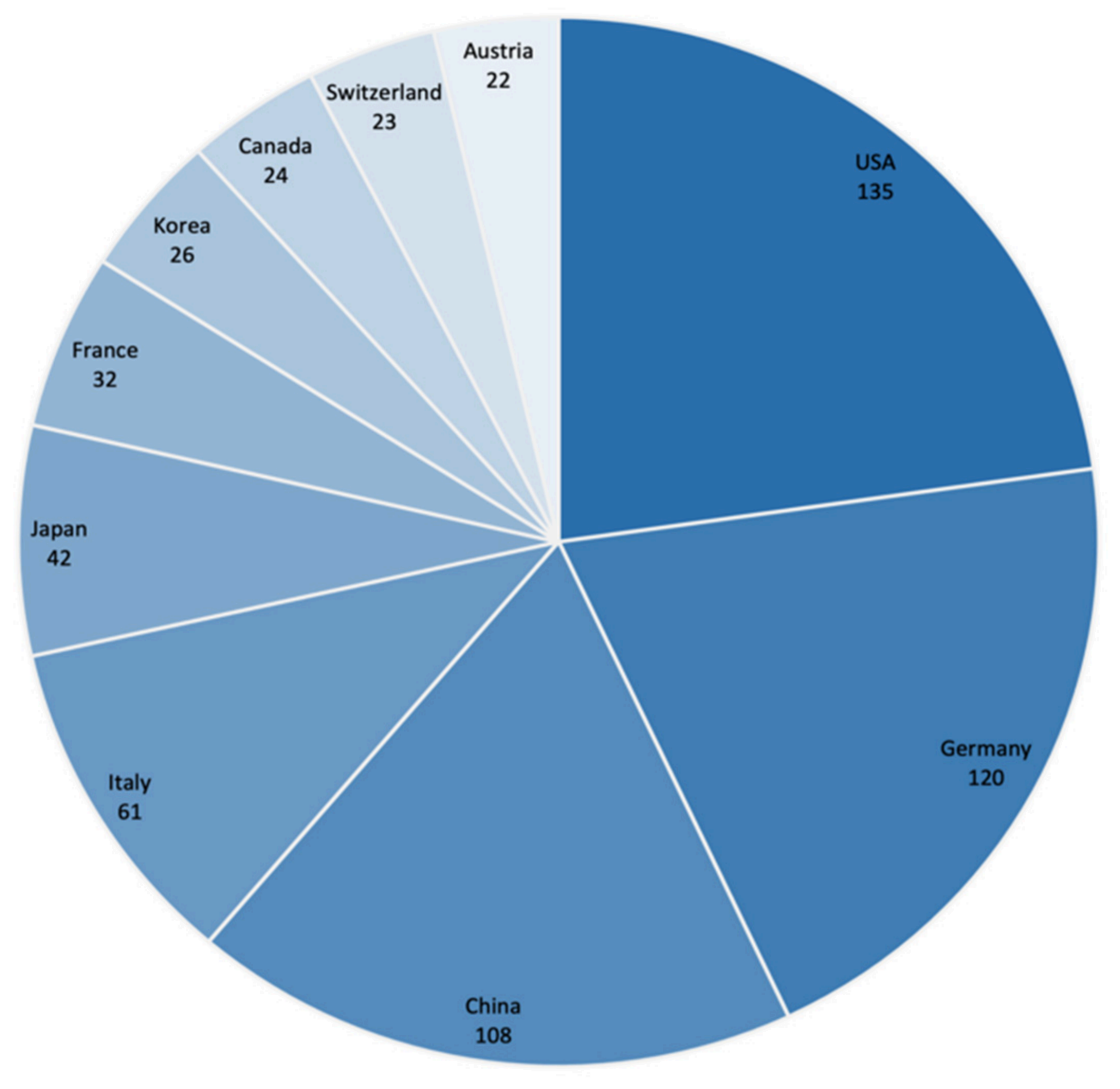
Top ten countries ranked by publication volume, showing absolute publication counts.

A total of 46 countries contributed to the dataset. The ten most productive countries accounted for 593 publications, representing 76.2% of the total output (Figure 3 (Table A1 for full country-level data), Figure 4). The United States led with 135 publications (17.4%), followed by Germany with 120 publications (15.4%) and China with 108 publications (13.9%). Egypt was the only African country represented (0.9%), and no other African country contributed to the dataset. Representation from South America and from most of Asia, outside China and Japan, was limited.

#### 3.2.4. Institutional Analysis

**Figure 5:**
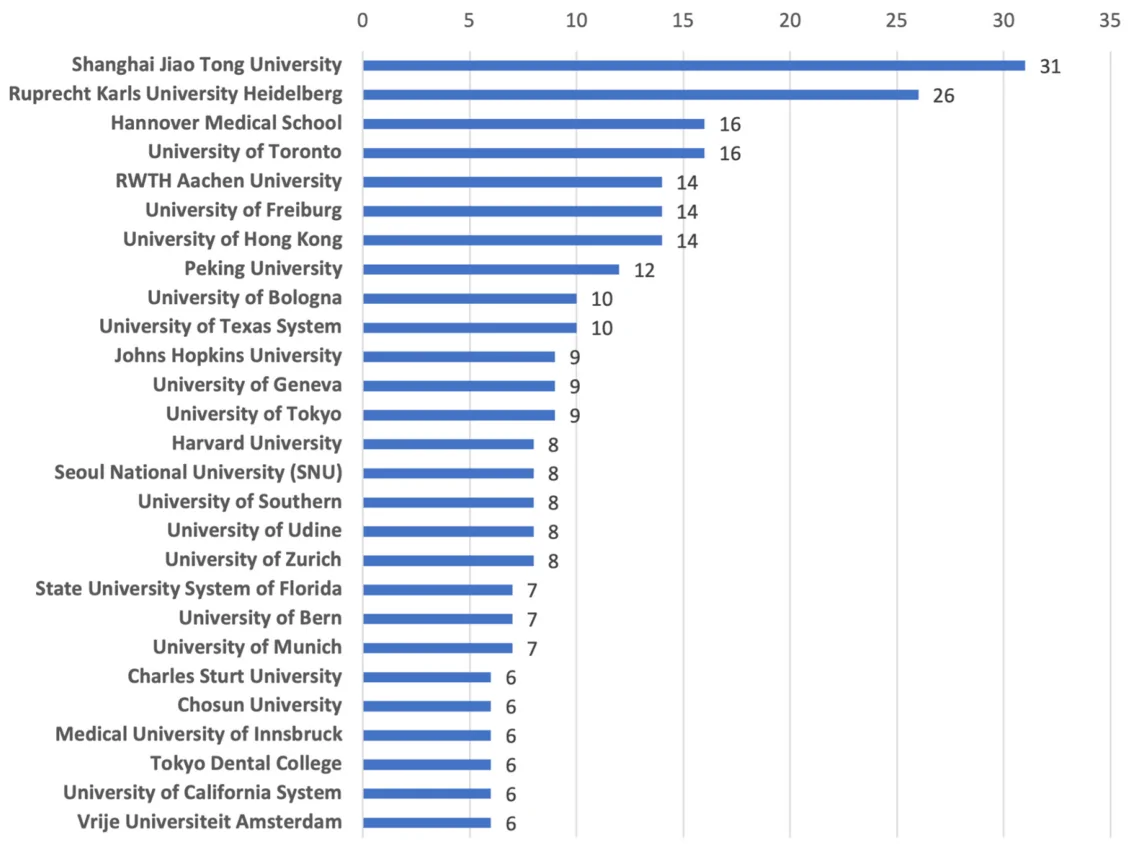
Leading academic institutions with six or more publications (*n* = 26), ranked by publication output.

Shanghai Jiao Tong University was the most productive institution, with 31 publications, followed by Ruprecht Karls University Heidelberg with 26. The University of Toronto and Hannover Medical School each contributed 16 publications (Figure 5). All leading institutions maintain active clinical surgical programs alongside their research activities.

#### 3.2.5. Author Productivity

**Figure 6:**
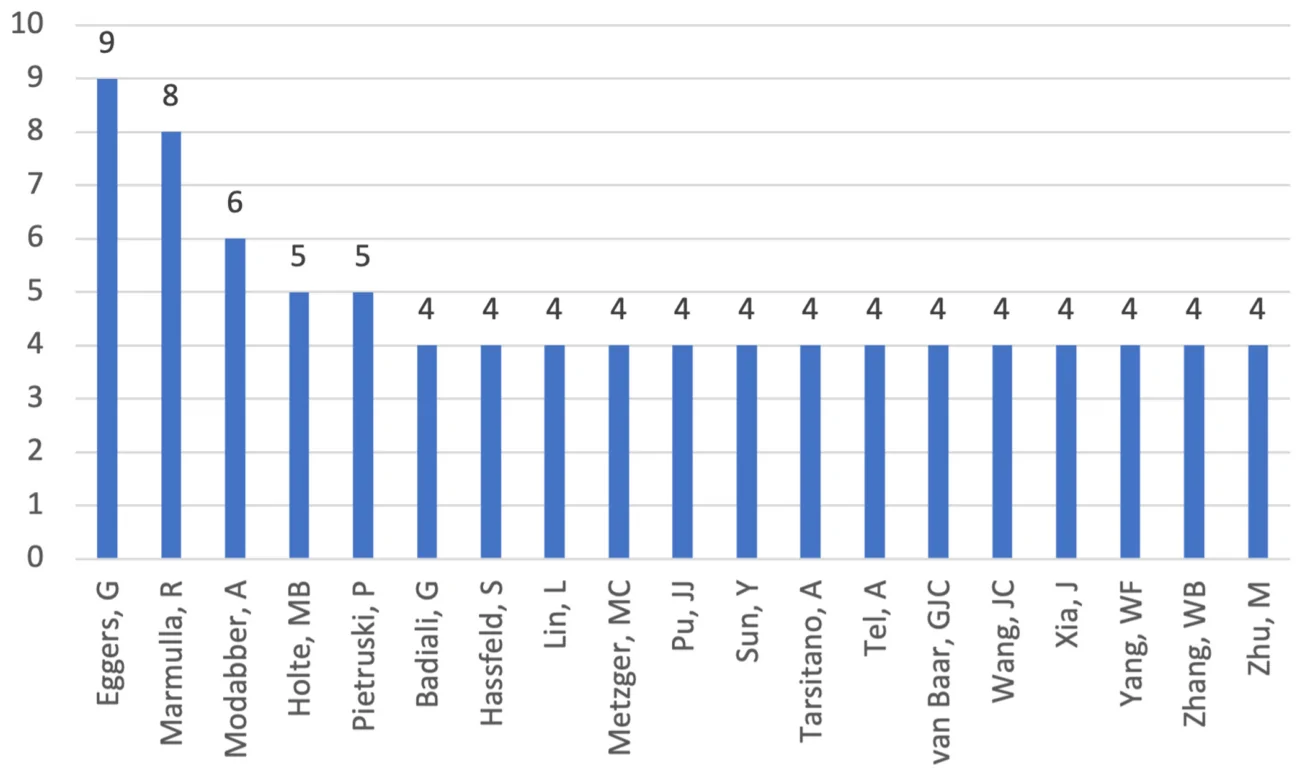
Most productive authors with four or more publications (*n* = 19) ranked by total publication output.

A total of 4463 authors contributed to the dataset. Publication output was concentrated among a small number of highly productive authors. Eggers G had the highest output, with nine publications, followed by Marmulla R with eight and Modabber A with six (Figure 6).

#### 3.2.6. Funding Landscape

Funding data were available for a subset of publications. The National Natural Science Foundation of China supported the largest number of funded studies (20 publications), followed by the Health and Medical Research Fund of Hong Kong (8 publications) and the National Institutes of Health in the USA (6 publications). Japanese agencies contributed one entry to five publications, and Projekt DEAL from Germany’s nationwide open access publishing agreement, which covers article processing charges rather than research funding, was linked to five publications. The Austrian Science Fund, Chosun University Dental Hospital, and the National Key R&D Program of China each supported four funded studies (Table 3).

### 3.3. Science Mapping

#### 3.3.1. Citation Analysis

**Figure 7:**
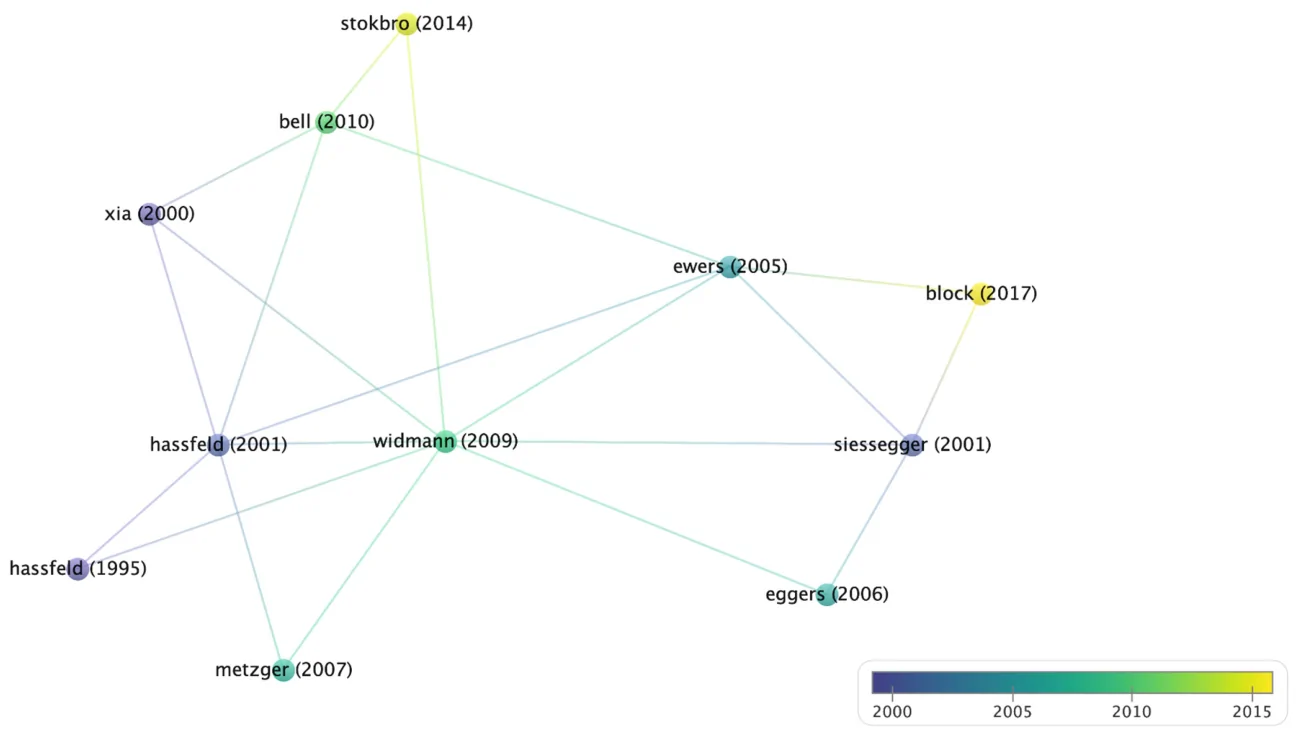
Citation network of highly cited publications (≥100 citations; 11 interconnected publications). Each node represents a publication labeled by primary author and year. Node color indicates publication year, ranging from purple (oldest) to yellow-green (most recent). Connecting lines indicate citation relationships.

Citation network analysis identified 26 articles with at least 100 citations, of which 11 were sufficiently interconnected to form a visualizable network (Figure 7). These 11 papers span 1995 to 2017 and are represented by a color gradient from purple (oldest) to yellow (most recent). The most connected node within the network was the 2005 review of navigation technology. The remaining 15 publications meet the citation threshold. The most globally cited publication in the dataset, the 2019 review of VR and AR in oral and maxillofacial surgery, does not appear in the network, as more recent publications have had less time to accumulate internal citations from other papers within the dataset.

At the journal level, the *Journal of Cranio-Maxillofacial Surgery* had the highest TLCS (339) and a TGCS of 1638, whereas the *International Journal of Oral and Maxillofacial Surgery* recorded the highest overall global citations (TGCS = 1824) with a TLCS of 318 (Table 4). *Applied Sciences—Basel* had a TLCS of 0, indicating that despite 14 publications in the dataset, none were cited by other papers in the dataset.

At the country level, the USA led TGCS (4398), while Germany led in TLCS (570), despite ranking second in publication volume (Table 5). Switzerland ranked sixth by publication count with TGCS of 1262 and TLCS of 146.

At the author level, Mühling J had the highest TLCS (171) and TGCS (883). Marchetti C had the highest TGCS per year (56.04) and Chai G the highest TLCS per year (11.14) (Table 6).

The five most cited individual publications spanned 2001 to 2019 (Table 7). The most cited was a 2019 review of VR and AR in oral and maxillofacial surgery ranked first with 216 WoS citations, followed by a 2001 review of computer-assisted OMS (201 citations), a 2005 review on navigation technology (199 citations), a 2017 study on dynamic navigation for implant placement (188 citations), and a 2014 paper on virtual planning in orthognathic surgery (181 citations). Full bibliographic details are provided in Table 7.

#### 3.3.2. Bibliographic Coupling

**Figure 8:**
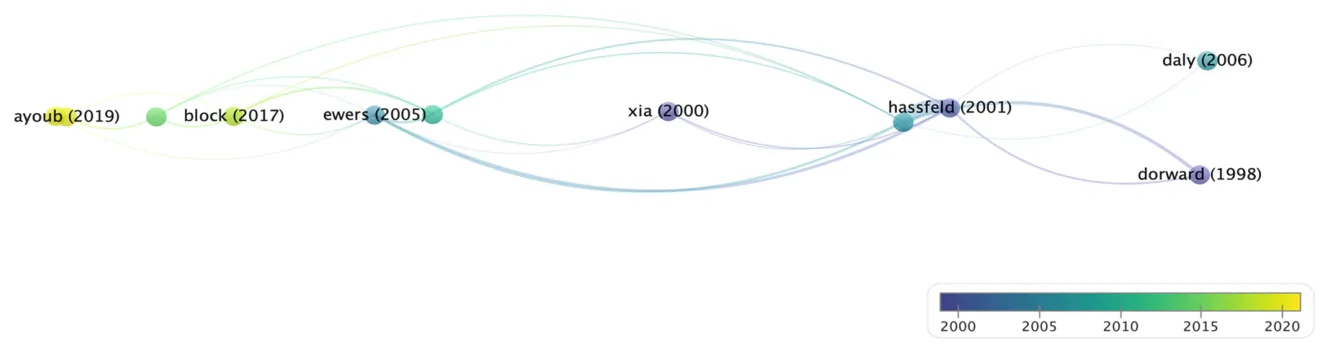
Bibliographic coupling network of publications meeting the citation threshold (≥150 citations; 7 interconnected publications). Each node represents a publication labeled by primary author and year. Node color indicates publication year, ranging from purple (oldest) to yellow-green (most recent). Connecting lines indicate shared references between publications.

Bibliographic coupling analysis applied a minimum citation threshold of 150, identifying seven interconnected publications (Figure 8). The network spans 1998 to 2019, with node color indicating publication year from purple (oldest) to yellow-green (most recent). The 2005 navigation-technology review occupies a central position with the highest number of connections. The most recent nodes, from 2017 and 2019, connect to older foundational works from 2000 and 2001, while the 1998 and 2006 nodes lie at the periphery of the network.

#### 3.3.3. Keyword Analysis

**Figure 9:**
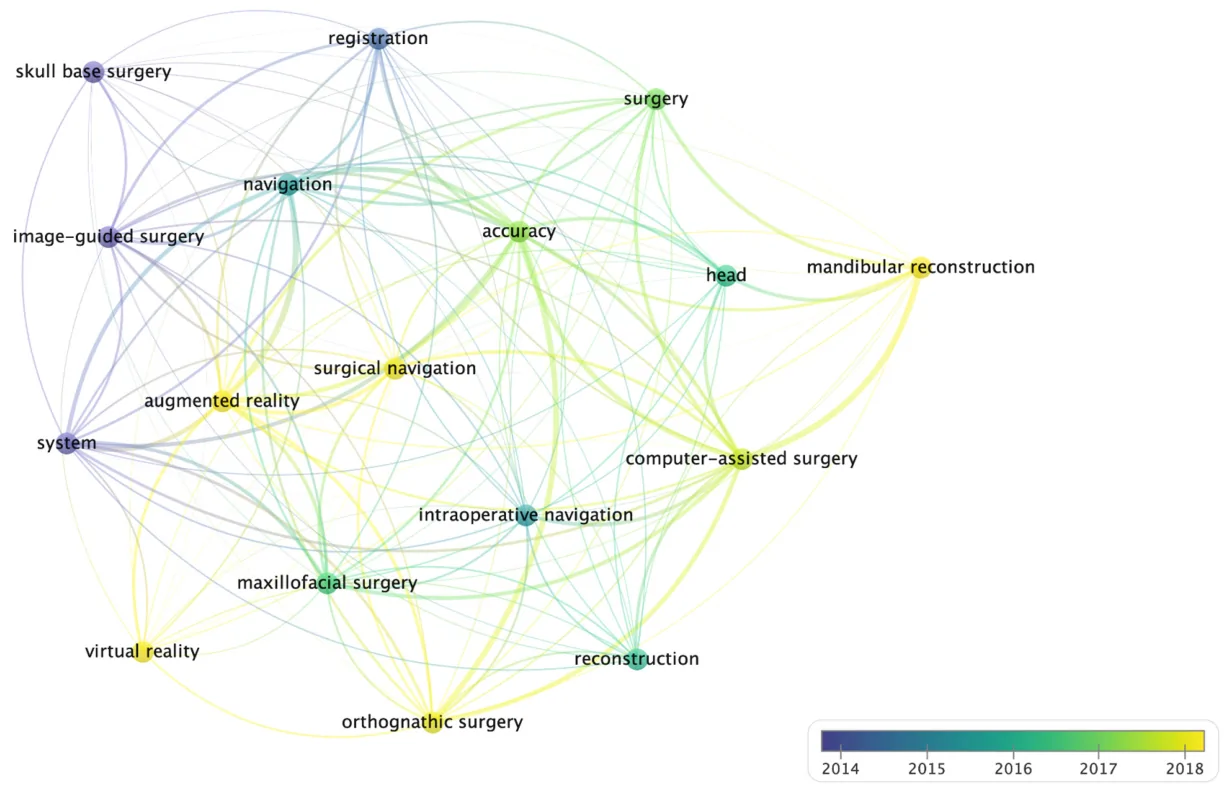
Keyword co-occurrence network of terms appearing at least 50 times across the dataset (17 terms). Node size reflects term frequency, and color indicates the average publication year of papers using each term, ranging from purple (~2014) to yellow-green (~2017–2018).

Keyword co-occurrence analysis identified 17 terms appearing at least 50 times across the dataset (Figure 9). Node color indicates the average publication year of papers using each term, ranging from purple (~2014) to yellow-green (~2017–2018). The network forms a single densely interconnected structure. Older terms include registration, navigation, image-guided surgery, and skull base surgery. Mid-period terms include augmented reality, surgical navigation, intraoperative navigation, computer-assisted surgery, and accuracy, among the most frequently used keywords. More recent terms include virtual reality, orthognathic surgery, reconstruction, and mandibular reconstruction. All 17 terms are connected to multiple other terms in the network.

#### 3.3.4. Co-Authorship Networks

**Figure 10:**
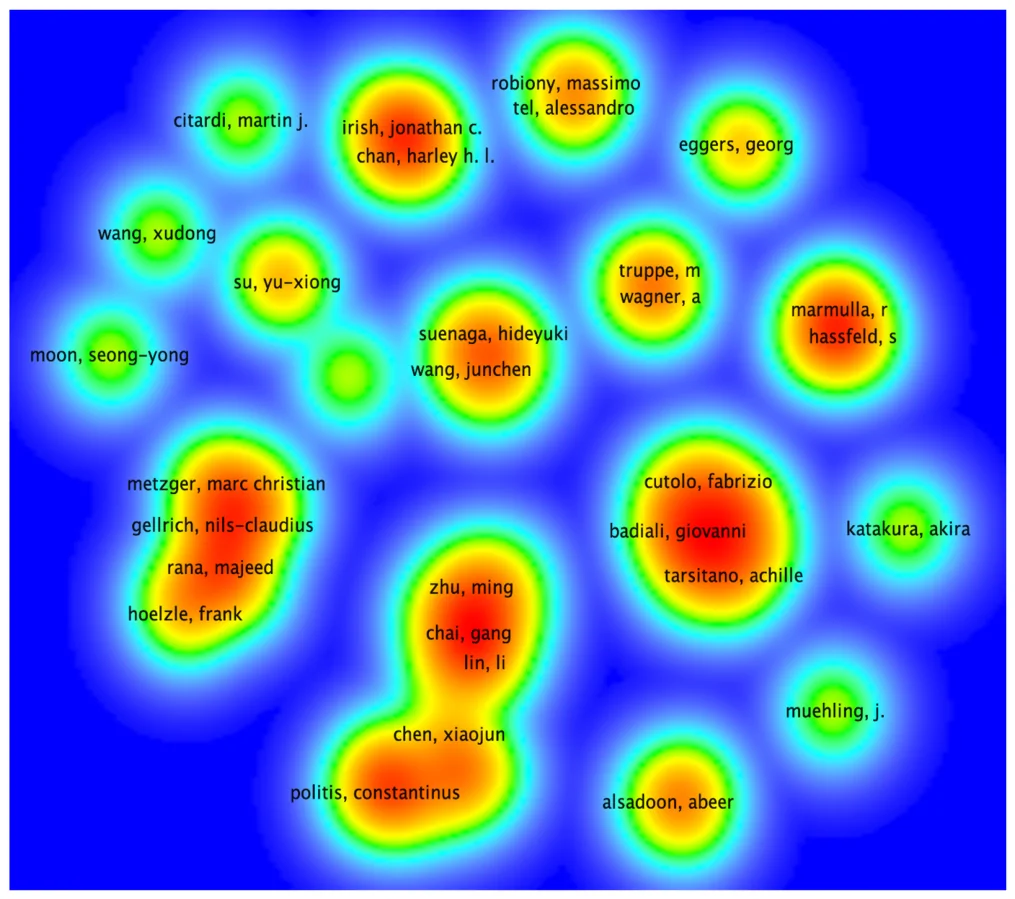
Author co-authorship density map (minimum threshold: five publications per author). Color intensity indicates co-authorship density, with red and orange denoting the highest-density zones and blue indicating minimal or no co-authorship activity.

Co-authorship analysis using a minimum threshold of five publications identified distinct collaboration clusters visualized as a density heatmap, with red and orange indicating the highest co-authorship density (Figure 10). Three primary high-density clusters are visible. The largest centers on Zhu Ming, Chai Gang, Lin Li, and Chen Xiaojun. A second cluster groups Irish JC, Chan HHL, and Citardi MJ. A third cluster includes Marmulla R and Hassfeld S. Additional moderate-density groupings are visible around Gellrich NC, Rana M, and Metzger MC; around Badiali G, Robiony M, and Tel A; and around Suenaga H and Truppe M. Limited overlap between clusters is evident across the map.

**Figure 11:**
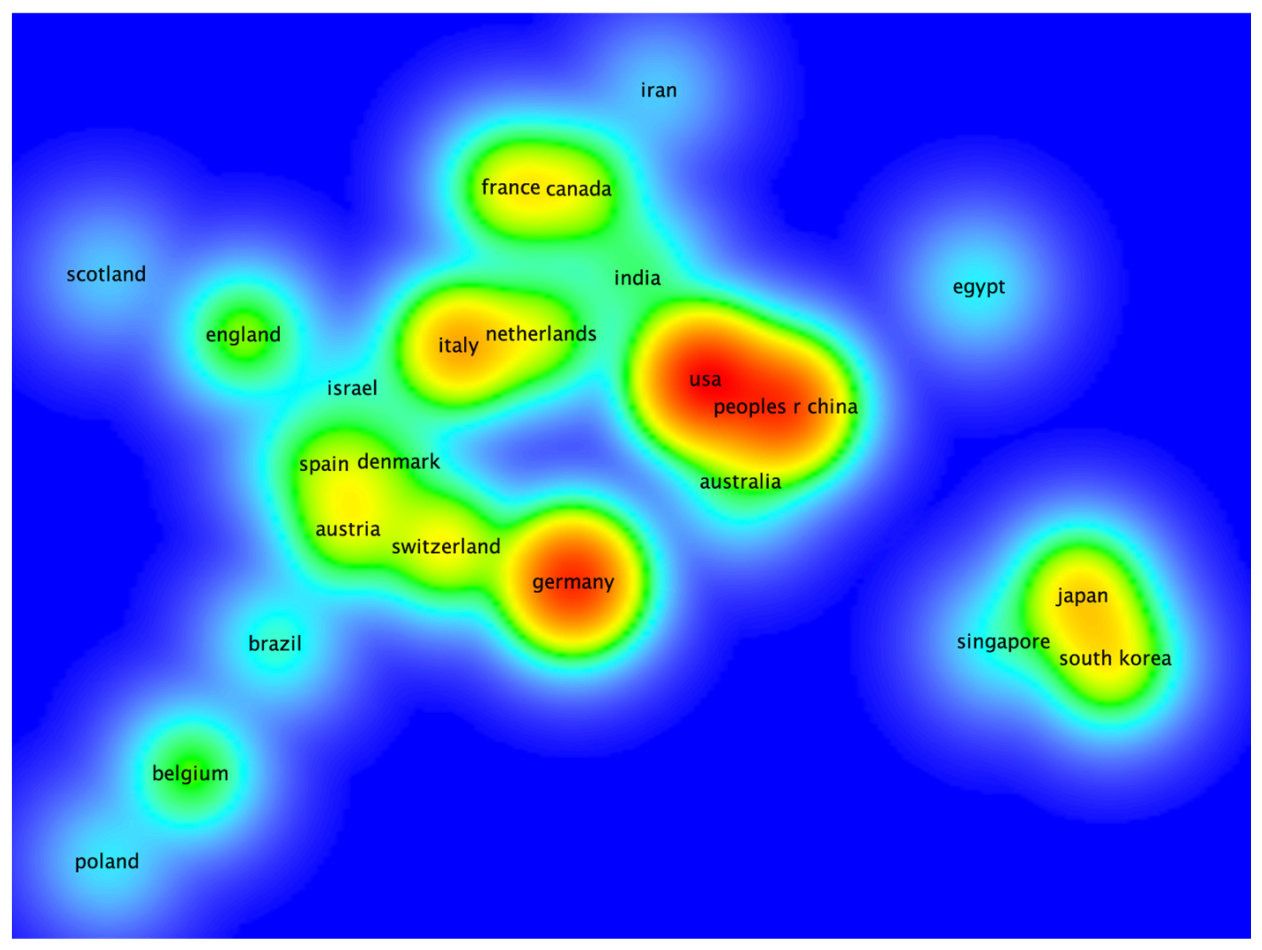
Country-level co-authorship density map (minimum threshold: five publications per author). Color intensity indicates collaboration density between countries, with red and orange representing the highest-density zones and blue representing minimal or no collaboration.

Country-level co-authorship analysis identified three dominant high-density collaboration zones (Figure 11). The largest centers are in the USA and China. The second group comprises Germany, with Austria and Switzerland in the connected zone. The third centers on Italy, with the Netherlands in the same zone. France and Canada form a moderate-density cluster linking the European zones. A separate cluster groups Japan, South Korea, and Singapore. England and Scotland form a smaller, independent cluster. Several countries, including Iran, India, Egypt, Israel, Brazil, Belgium, Poland, Spain, and Denmark, appear as peripheral nodes with limited collaboration activity. Egypt was the only African country visible in the network, appearing as a peripheral node with limited collaboration activity.

## 4. Discussion

The growth in XR and computer-assisted surgery research in CMF surgery, particularly the acceleration observed after 2010, aligns with broader advances in computing power, display technologies, and medical imaging, which have made clinical implementation increasingly feasible [25,26]. The field has evolved from early experimental applications to an established research domain, yet output remains concentrated among a relatively small number of highly productive researchers, leaving room for broader community participation.

The concentration of publications in specialist OMS and CMF surgery journals, rather than in engineering or computer science outlets, is consistent with a field in which clinical application has been a primary driver of research. The contrasting case of *Applied Sciences—Basel* is instructive in this regard. Despite contributing 14 publications to the dataset, it recorded a TLCS of zero, meaning none of those publications were cited by other papers within this literature. Work published outside specialist surgical venues appears not to enter the citation networks of the CMF surgical community, suggesting that visibility within the field depends heavily on choice of publication venue.

The geographic concentration of output in the USA, Germany, and China reflects differences in research infrastructure, funding availability, and institutional capacity for interdisciplinary work [27]. Within this concentration, however, Germany’s citation profile is notable. Despite ranking second in publication volume, it leads in TLCS with a score of 570, compared with the USA’s 281, indicating that German research exerts disproportionate influence within the CMF XR community relative to its output volume. Switzerland shows a similar pattern at a smaller scale, ranking sixth by publication count yet achieving citation yields comparable to those of higher-volume countries. These findings suggest that research intensity and specialization matter alongside volume. The near-complete absence of African contributions, except for a single country, Egypt, and limited output from South America and most of Asia raises concerns about equitable access to XR technology development and the generalizability of findings to diverse healthcare settings [28]. Whether technologies validated predominantly in high-resource environments translate to lower-resource contexts remains an open question that the current literature does not address.

The leading institutions in this field, including Shanghai Jiao Tong University and Ruprecht Karls University Heidelberg, maintain active clinical surgical programs alongside technical research capacity. This pattern suggests that productive XR research in CMF surgery tends to emerge from environments where surgical expertise and engineering or computational resources coexist. Notably, the inclusion of Chosun University Dental Hospital among the top funding sources is significant, as institutional-level funding from a specialist clinical center, rather than from a national research council, suggests that hospital-based research units are playing a meaningful role in driving this field forward alongside national funding bodies.

Citation analysis reveals three broadly identifiable phases in the development of this field. The foundational phase (1990s–2000s), represented by highly cited publications in the dataset, including the 2001 review of computer-assisted oral and maxillofacial surgery and the 2005 review of navigation technology, centered on establishing computer-assisted navigation and image-guided surgery as viable approaches in CMF practice. A developmental phase (2000s–2010s) consolidated these foundations and expanded applications across orthognathic surgery, implant placement, and reconstructive planning, as reflected in the 2014 study of virtual planning in orthognathic surgery and the 2017 study of dynamic navigation for implant placement. The most recent phase (2010s–present) has shifted towards optimizing clinical application, specifically the integration of AR and VR, with the 2019 review of VR and AR representing the most globally cited contribution from this period. This three-phase progression is independently supported by keyword temporal patterns, in which older terms, including registration, navigation, and image-guided surgery, cluster in earlier publications, while virtual reality, orthognathic surgery, and mandibular reconstruction emerge as more recent dominant terms, reflecting the gradual shift in research vocabulary alongside the underlying technological transition. The bibliographic coupling network reinforces this narrative continuity, with the most recent nodes (2017 and 2019) sharing reference relationships with foundational works from 2000 and 2001, indicating that modern XR research continues to build on two-decade-old methodological foundations rather than representing a clean break from the computer-assisted surgery era.

The keyword network mirrors this progression. The oldest terms name the enabling technology itself (image-guided surgery, registration, skull base surgery), while AR and VR applications have emerged more recently alongside specific procedures such as orthognathic surgery and mandibular reconstruction, with the literature still weighted toward feasibility studies rather than clinical outcome trials. Accuracy occupies a central, bridging position in the network, connecting the older navigation cluster to the newer XR cluster, suggesting it has remained the field’s persistent evaluative measure across this transition. Citation centrality and publication recency within an application area thus offer a practical indicator of relative evidence maturity, not of clinical benefit.

Despite substantial output, the most highly cited publications in this dataset are predominantly reviews and methodological studies rather than clinical outcome trials. This pattern may suggest that clinical outcome evidence accounts for a comparatively smaller share of the most influential literature in this field, though bibliometric data alone cannot confirm this and would require dedicated evidence synthesis to verify. The strong regional clustering visible in the co-authorship networks, with limited cross-cluster connectivity between the major groups, indicates that knowledge exchange between research communities remains constrained, which may slow the development of consistent evaluation approaches across the field. Funding concentration in a small number of countries further narrows the diversity of research priorities.

The keyword analysis identifies several areas where research activity is relatively recent and still developing, including virtual reality, mandibular reconstruction, and orthognathic surgery. This recency may indicate that comparative clinical evidence in these areas is still emerging, though keyword patterns alone cannot directly measure the maturity of the underlying evidence base. As XR becomes increasingly integrated into broader digital surgical workflows alongside CAD-CAM technologies, point-of-care manufacturing, and robotic-assisted surgery, the absence of consistent outcome reporting and comparative effectiveness represents a significant gap for institutions considering adoption [29,30,31,32,33].

This study has several limitations. The analysis was restricted to English-language publications indexed in WoS, which may underrepresent research from non-English-speaking countries and from journals not covered by this database. The bibliometric approach captures publication and citation patterns but cannot assess research quality, clinical validity, or patient outcomes. Finally, the rapid pace of technological development means that recent innovations may not yet be fully visible in the literature at the time of data extraction.

Future research priorities should include expanding international collaboration, particularly with institutions in underrepresented regions, to improve access to technology and address existing geographic disparities. Greater focus on patient-reported outcomes and cost-effectiveness analyses would strengthen the clinical evidence base and support translation into routine surgical practice. Cross-cluster collaboration among currently isolated regional research groups offers a concrete opportunity to accelerate knowledge exchange. Longitudinal studies tracking adoption patterns across different healthcare system contexts would also help clarify which XR applications offer the greatest benefit compared with existing planning and surgical approaches.

## 5. Conclusions

This analysis maps 36 years of XR and computer-assisted surgery research in CMF surgery across 778 publications, identifying three broad developmental phases, from early computer-assisted navigation to contemporary AR and VR applications. Research output is geographically concentrated, with strong influence from a small number of countries and institutions. The near-complete absence of African contributions highlights significant global disparities. The most cited literature remains dominated by reviews and methodological studies rather than clinical outcome trials, which may imply that clinical outcome evidence occupies a comparatively smaller share of the most influential literature in this field, an inference that bibliometric data alone cannot confirm. As XR becomes integrated into broader digital surgical workflows, addressing gaps in clinical evidence, consistent outcome reporting, and international collaboration will be essential to translate this field’s research investment into wider surgical practice.

## Figures and Tables

**Table 1:** Parameters used in citation analysis with corresponding descriptions.

Parameter	Description
TLCS (Total Local Citation Score)	Indicates the number of times other documents within the same dataset have cited a publication and measures its impact within the dataset.
TLCS/t	Indicates the TLCS per year.
TLCSx (Total Local Citation Score excluding self-citations)	Indicates the number of times other documents within the same dataset have cited a publication without counting the author’s self-citations.
TGCS (Total Global Citation Score)	Indicates the number of times a publication has been cited in the database. It reflects the global impact of the publication across all disciplines and journals.
TGCS/t	Indicates the TGCS per year.
TLCR (Total Local Citation References)	Represents the number of references in a publication that cites other documents within the same dataset.

**Table 2:** Top ten journals ranked by publication volume with corresponding 5-year impact factor (2025).

S. No.	Journal	Number of Publications (%)	5-Year IF (2025)
1	*Journal of Cranio-Maxillofacial Surgery*	56	2.5
2	*International Journal of Oral and Maxillofacial Surgery*	47	2.6
3	*Journal of Craniofacial Surgery*	46	1.1
4	*International Journal of Computer Assisted Radiology and Surgery*	25	3.1
5	*Otolaryngology—Head and Neck Surgery*	24	3.3
6	*Journal of Oral and Maxillofacial Surgery*	21	2.1
7	*International Journal of Medical Robotics and Computer Assisted Surgery*	20	2.7
8	*British Journal of Oral & Maxillofacial Surgery*	16	1.5
9a	*Applied Sciences—Basel*	14	2.7
9b	*Laryngoscope*	14	2.6
10	*Journal of Clinical Medicine*	13	3.4

**Table 3:** Leading funding agencies ranked by number of funded publications.

S. No.	Funding Agency	Number of Publications
1	National Natural Science Foundation of China	20
2	Health and Medical Research Fund, Hong Kong	8
3	National Institutes of Health, USA	6
4a	Japan Society for the Promotion of Science *	5
4b	Projekt DEAL, Germany ^†^	5
5a	Austrian Science Fund	4
5b	Chosun University Dental Hospital	4
5c	National Key R&D Program of China **	4

* WoS indexes funding sources as reported by authors. Publications indexed under “JSPS KAKENHI” and “Grants-in-Aid for Scientific Research, Japan” are captured in this entry as they refer to the same program. ^†^ Projekt DEAL is a German nationwide open access publishing agreement between research institutions and academic publishers; it covers article processing charges rather than research funding, and its inclusion here reflects WoS indexing of publication-cost support rather than direct research sponsorship. ** Publications indexed under “National Key Research and Development Program of China” are captured within this entry.

**Table 4:** Top ten journals ranked by citation metrics.

S. No.	Journal	TLCS	TLCS/t	TGCS	TGCS/t	TLCR
1	*Journal of Cranio-Maxillofacial Surgery*	339	31.21	1638	147.11	153
2	*International Journal of Oral and Maxillofacial Surgery*	318	26.73	1824	141.69	170
3	*Journal of Craniofacial Surgery*	128	11.20	803	69.08	126
4	*International Journal of Computer Assisted Radiology and Surgery*	56	6.07	458	49.11	52
5	*Otolaryngology—Head and Neck Surgery*	54	3.58	621	39.34	27
6	*Journal of Oral and Maxillofacial Surgery*	78	4.50	453	33.60	54
7	*International Journal of Medical Robotics and Computer Assisted Surgery*	37	2.36	599	50.28	53
8	*British Journal of Oral and Maxillofacial Surgery*	62	5.14	314	26.97	27
9	*Applied Sciences—Basel*	0	0.00	122	26.10	28
10	*Laryngoscope*	53	5.77	373	35.03	51

TLCS = Total Local Citation Score; TLCS/t = TLCS per year; TGCS = Total Global Citation Score; TGCS/t = TGCS per year; TLCR = Total Local Citation References.

**Table 5:** Country-level citation impact analysis.

S. No.	Country	TGCS	TLCS
1	USA	4398	281
2	Germany	4033	570
3	People’s Republic China	2345	323
4	Italy	1309	151
5	Japan	1086	109
6	Switzerland	1262	146
7	France	691	50
8	UK	1262	65
9	Austria	1102	152

TGCS = Total Global Citation Score; TLCS = Total Local Citation Score.

**Table 6:** Most influential authors ranked by bibliometric indicators.

S. No.	Author	TLCS	TLCS/t	TLCSx	TGCS	TGCS/t	TLCR
1	Mühling J	171	8.12	135	883	42.16	64
2	Gellrich NC	82	5.69	63	643	44.58	67
3	Chai G	101	11.14	73	396	48.83	63
4	Irish JC	51	3.73	24	509	38.13	47
5	Marmulla R	114	5.40	88	559	26.84	44
6	Eggers G	103	5.41	82	500	26.74	46
7	Rana M	41	3.31	26	375	34.92	42
8	Marchetti C	110	11.93	86	454	56.04	48
9	Badiali G	82	8.26	66	352	50.27	57
10	Daly MJ	48	3.56	24	470	35.24	41

TLCS = Total Local Citation Score; TLCS/t = TLCS per year; TLCSx = TLCS excluding self-citations; TGCS = Total Global Citation Score; TGCS/t = TGCS per year; TLCR = Total Local Citation References.

**Table 7:** Most cited publications ranked by citation count.

S. No.	Author	Title	Publication Year	Citations (WoS)	Citations in Databases	References
1	Ayoub, A	The application of virtual reality and augmented reality in Oral & Maxillofacial Surgery	November 2019	216	232	50
2	Hassfeld, S	Computer assisted oral and maxillofacial surgery: a review and an assessment of technology	February 2001	201	227	138
3	Ewers, R	Basic research and 12 years of clinical experience in computer-assisted navigation technology: a review	January 2005	199	216	36
4	Block, MS	Implant Placement Accuracy Using Dynamic Navigation	January 2017	188	207	39
5	Stokbro, K	Virtual planning in orthognathic surgery	August 2014	181	184	50

## Data Availability

The datasets of this study are available from the corresponding author upon reasonable request.

## Abbreviations

2D	Two-dimensional
3D	Three-dimensional
AR	Augmented Reality
CAD	Computer-Aided Design
CAM	Computer-Aided Manufacturing
CMF	Craniomaxillofacial Surgery
MR	Mixed Reality
TLCS	Total Local Citation Score
TLCS/t	Total Local Citation Score per year
TLCSx	Total Local Citation Score excluding self-citations
TGCS	Total Global Citation Score
TGCS/t	Total Global Citation Score per year
TLCR	Total Local Citation References
VR	Virtual Reality
WoS	Web of Science
XR	Extended Reality

## Appendix A

**Table A1:** Full country-level distribution of publication output (*n* = 778 records across 46 countries), Web of Science Core Collection, 1988–2024. Countries are ranked by publication count, corresponding to the shading classes used in Figure 3.

S. No.	Country	Publications (*n*)	Share of Total (%)
1	United States	135	17.4
2	Germany	120	15.4
3	China	108	13.9
4	Italy	61	7.8
5	Japan	42	5.4
6	France	32	4.1
7	South Korea	26	3.3
8	Canada	24	3.1
9	Switzerland	23	3.0
10	Austria	22	2.8
11	Netherlands	22	2.8
12	United Kingdom	21	2.7
13	Belgium	15	1.9
14	Spain	12	1.5
15	Australia	9	1.2
16	Turkey	9	1.2
17	Taiwan	8	1.0
18	Singapore	8	1.0
19	Poland	7	0.9
20	Egypt	7	0.9
21	Brazil	6	0.8
22	Denmark	6	0.8
23	Sweden	6	0.8
24	India	6	0.8
25	Saudi Arabia	5	0.6
26	Iran	4	0.5
27	Thailand	4	0.5
28	Romania	3	0.4
29	Israel	3	0.4
30	Argentina	2	0.3
31	Malaysia	2	0.3
32	Russia	2	0.3
33	Cyprus	2	0.3
34	Chile	2	0.3
35	Norway	2	0.3
36	Mexico	2	0.3
37	Finland	1	0.1
38	Croatia	1	0.1
39	Philippines	1	0.1
40	Pakistan	1	0.1
41	Oman	1	0.1
42	Lithuania	1	0.1
43	Greece	1	0.1
44	Kuwait	1	0.1
45	Czech Republic	1	0.1
46	Ukraine	1	0.1
